# Supplementary material for: Virtual serious games for women’s health education: A scoping review
Source: PLoS One. 2025 Jun 3;20(6):e0325327. doi: 10.1371/journal.pone.0325327 (PMC12133008; doi:10.1371/journal.pone.0325327)
Supplement: S1 File — (PDF) [file pone.0325327.s001.pdf]

S1 File. Search strategy

MEDLINE (Ovid)

Search conducted on April 22, 2024; 1006 citations found

#	Searches	Results
1	((game* not gamete*) or videogame* or gaming or VSG*).mp.	71426
2	Video Games/	7433
3	Games, Experimental/	2357
4	or/1-3	71428
5	((patient* or client* or consumer* or health*) adj3 (educat* or communicat* or knowledge* or learn* or understand* or inform* or activit* or engag* or participat* or program* or teach* or promot* or litera*)).mp.	998618
6	Patient Education as Topic/	88463
7	Health Communication/	3363
8	Consumer Health Information/	4336
9	Health Knowledge, Attitudes, Practice/	128182
10	Health Promotion/	82547
11	Health Literacy/	9852
12	Patient Participation/	29860
13	or/5-12	998618
14	(Wom?n* or mother* or matern* or female*).mp.	10266367
15	Women/	15207
16	14 or 15	10266367
17	4 and 13 and 16	1693
18	limit 17 to "all adult (19 plus years)"	829
19	limit 17 to "all child (0 to 18 years)"	982
20	17 not 19	711
21	18 or 20	1046
22	limit 21 to english language	1006

CINHAL

Search conducted on April 22, 2024; 219 citations found

#	Query	Limiters/Expanders	Last Run Via	Results
S20	S19	*Limiters - English Language*<br>*Expanders - Apply related words; Apply equivalent subjects*<br>*Search modes - Boolean/Phrase*	*Interface - EBSCOhost Research Databases*<br>*Search Screen - Advanced Search*<br>*Database - CINAHL Plus with Full Text*	219
S19	S16 OR S18	*Expanders - Apply related words; Apply equivalent subjects*<br>*Search modes - Boolean/Phrase*	*Interface - EBSCOhost Research Databases*<br>*Search Screen - Advanced Search*<br>*Database - CINAHL Plus with Full Text*	221
S18	S15 NOT S17	*Expanders - Apply related words; Apply equivalent subjects*<br>*Search modes - Boolean/Phrase*	*Interface - EBSCOhost Research Databases*<br>*Search Screen - Advanced Search*<br>*Database - CINAHL Plus with Full Text*	177
S17	S15	*Limiters - Age Groups: Infant, Newborn: birth-1 month, Infant: 1-23 months, Child, Preschool: 2-5 years, Child: 6-12 years, Adolescent: 13-18 years*<br>*Expanders - Apply related words; Apply equivalent subjects*<br>*Search modes - Boolean/Phrase*	*Interface - EBSCOhost Research Databases*<br>*Search Screen - Advanced Search*<br>*Database - CINAHL Plus with Full Text*	160
S16	S15	*Limiters - Age Groups: Adult: 19-44 years, Middle Aged: 45-64 years, Aged: 65+ years, Aged, 80 and over*<br>*Expanders - Apply related words; Apply equivalent subjects*<br>*Search modes - Boolean/Phrase*	*Interface - EBSCOhost Research Databases*<br>*Search Screen - Advanced Search*<br>*Database - CINAHL Plus with Full Text*	178
S15	*S3 AND S11 AND S14*	*Expanders - Apply related words; Apply equivalent subjects*<br>*Search modes - Boolean/Phrase*	*Interface - EBSCOhost Research Databases*<br>*Search Screen - Advanced Search*<br>*Database - CINAHL Plus with Full Text*	337
S14	*S12 OR S13*	*Expanders - Apply related words; Apply equivalent subjects*<br>*Search modes - Boolean/Phrase*	*Interface - EBSCOhost Research Databases*<br>*Search Screen - Advanced Search*<br>*Database - CINAHL Plus with Full Text*	*2,479,594*
S13	*(MH "Women")*	*Expanders - Apply related words; Apply equivalent subjects*<br>*Search modes - Boolean/Phrase*	*Interface - EBSCOhost Research Databases*<br>*Search Screen - Advanced Search*<br>*Database - CINAHL Plus with Full Text*	*26,976*
S12	*(Wom?n* or mother* or matern* or female*)*	*Expanders - Apply related words; Apply equivalent subjects*<br>*Search modes - Boolean/Phrase*	*Interface - EBSCOhost Research Databases*<br>*Search Screen - Advanced Search*	*2,479,594*

			Database - CINAHL Plus with Full Text	
S11	*S4 OR S5 OR S6 OR S7 OR S8 OR S9 OR S10*	*Expanders - Apply related words; Apply equivalent subjects Search modes - Boolean/Phrase*	*Interface - EBSCOhost Research Databases Search Screen - Advanced Search Database - CINAHL Plus with Full Text*	*688,123*
S10	*(MH "Consumer Participation")*	*Expanders - Apply related words; Apply equivalent subjects Search modes - Search modes - Boolean/Phrase*	*Interface - EBSCOhost Research Databases Search Screen - Advanced Search Database - CINAHL Plus with Full Text*	*24,694*
S9	*(MH "Health Literacy")*	*Expanders - Apply related words; Apply equivalent subjects Search modes - Boolean/Phrase*	*Interface - EBSCOhost Research Databases Search Screen - Advanced Search Database - CINAHL Plus with Full Text*	*7,538*
S8	*MH "Health Promotion")*	*Expanders - Apply related words; Apply equivalent subjects Search modes - Boolean/Phrase*	*Interface - EBSCOhost Research Databases Search Screen - Advanced Search Database - CINAHL Plus with Full Text*	*80,546*
S7	*(MH "Health Knowledge") Expanders*	*Expanders - Apply related words; Apply equivalent subjects Search modes - Boolean/Phrase*	*Interface - EBSCOhost Research Databases Search Screen - Advanced Search Database - CINAHL Plus with Full Text*	*39,452*
S6	*(MH "Consumer Health Information")*	*Expanders - Apply related words; Apply equivalent subjects Search modes - Boolean/Phrase*	*Interface - EBSCOhost Research Databases Search Screen - Advanced Search Database - CINAHL Plus with Full Text*	*14,006*
S5	*(MH "Patient Education")*	*Expanders - Apply related words; Apply equivalent subjects Search modes - Boolean/Phrase*	*Interface - EBSCOhost Research Databases Search Screen - Advanced Search Database - CINAHL Plus with Full Text*	*72,063*
S4	*((patient* or client* or consumer* or health*) N3 (educat* or communicat* or knowledge* or learn* or understand* or inform* or activit* or engag* or participat* or program* or teach* or promot* or litera*))*	*Expanders - Apply related words; Apply equivalent subjects Search modes - Boolean/Phrase*	*Interface - EBSCOhost Research Databases Search Screen - Advanced Search Database - CINAHL Plus with Full Text*	*688,123*

S3	*S1 OR S2*	*Expanders - Apply related words; Apply equivalent subjects*<br>*Search modes - Boolean/Phrase*	*Interface - EBSCOhost Research Databases*<br>*Search Screen - Advanced Search*<br>*Database - CINAHL Plus with Full Text*	*8,236*
S2	*(MH "Video Games")*	*Expanders - Apply related words; Apply equivalent subjects*<br>*Search modes - Boolean/Phrase*	*Interface - EBSCOhost Research Databases*<br>*Search Screen - Advanced Search*<br>*Database - CINAHL Plus with Full Text*	*5,746*
S1	*((game* not gamete*) or videogame* or gaming or VSG*)*	*Expanders - Apply related words; Apply equivalent subjects*<br>*Search modes - SmartText Searching*	*Interface - EBSCOhost Research Databases*<br>*Search Screen - Advanced Search*<br>*Database - CINAHL Plus with Full Text*	4

Embase (Ovid)

Search conducted on April 22, 2024; 1903 citations found

#	Searches	Results
1	((game* not gamete*) or videogame* or gaming or VSG*).mp.	85077
2	Video Game/	6453
3	Game/	9249
4	or/1-3	85084
5	((patient* or client* or consumer* or health*) adj3 (educat* or communicat* or knowledge* or learn* or understand* or inform* or activit* or engag* or participat* or program* or teach* or promot* or litera*)).mp.	1383256
6	Patient Education/	128364
7	Medical Information/	90955
8	Consumer Health Information/	4325
9	Attitude to Health/	133921
10	Health Promotion/	114348
11	Health Literacy/	21332
12	Patient Participation/	37242
13	or/5-12	1524370
14	(Wom?n* or mother* or matern* or female*).mp.	12621405
15	Female/	11927258
16	14 or 15	12621405
17	4 and 13 and 16	2936
18	limit 17 to adult <18 to 64 years>	1475
19	limit 17 to (infant or child or preschool child <1 to 6 years> or school child <7 to 12 years> or adolescent <13 to 17 years>)	1388
20	17 not 19	1548
21	18 or 20	1947
22	limit 21 to english language	1903

PsycInfo (Ovid)

Search conducted on April 22, 2024; 472 citations found

#	Searches	Results
1	((game* not gamete*) or videogame* or gaming or VSG*).mp.	62422
2	Computer Games/	9849
3	Games/	13009
4	or/1-3	62425
5	((patient* or client* or consumer* or health*) adj3 (educat* or communicat* or knowledge* or learn* or understand* or inform* or activit* or engag* or participat* or program* or teach* or promot* or litera*)).mp.	298412
6	Patient Education/	4782
7	Health Education/	15315
8	Health Information/	3695
9	Health Knowledge/	9743
10	Health Promotion/	29591
11	Health Literacy/	4385
12	Client Participation/	3368
13	or/5-12	298412
14	(Wom?n* or mother* or matern* or female*).mp.	1426126
15	Human Females/	99292
16	14 or 15	1426126
17	4 and 13 and 16	680
18	limit 17 to "300 adulthood "	425
19	limit 17 to (100 childhood or 120 neonatal or 140 infancy <2 to 23 mo> or 160 preschool age or 180 school age or 200 adolescence)	311
20	17 not 19	369
21	18 or 20	490
22	limit 21 to english language	472

Web of Science

Search conducted on April 22, 2024; 598 citations found

((game not gamete*) or videogame* or gaming or VSG*) AND ((patient* or client* or consumer* or health*) NEAR/3 (educat* or communicat* or knowledge* or learn* or understand* or inform* or activit* or engag* or participat* or program* or teach* or promot* or litera*)) AND (Wom?n* or mother* or matern* or female*)*

(Search by topic)
