# Supplementary material for: Virtual serious games for women’s health education: A scoping review
Source: PLoS One. 2025 Jun 3;20(6):e0325327. doi: 10.1371/journal.pone.0325327 (PMC12133008; doi:10.1371/journal.pone.0325327)
Supplement: S2 File — (PDF) [file pone.0325327.s002.pdf]

S2 File. Studies ineligible following full-text review

1. Angles Acedo S, Kastelein AW, Ros Cerro C, Raatikainen K, Alonso Lopez JF, Pages Raventos A, et al. Information and Communication Technologies (ICT) self management system for pelvic floor muscle training: A pilot study in women with stress urinary incontinence. International Urogynecology Journal. 2018;29(Supplement 1):S75.
   Reason for exclusion: Ineligible source.
2. Byrne S, Robles-Rodriguez E. Educational parties as a strategy to promote breast health awareness and screening in underserved female populations. Oncol Nurs Forum. 2009;36(2):145-8.
   Reason for exclusion: Ineligible intervention.
3. De Deugd N, Hage R. Personalized nutrition intervention: Machine-learning algorithm at the intersection of gaming, medicine and behavior change. Global Advances in Health and Medicine. 2020;9:133.
   Reason for exclusion: Ineligible source.
4. Derksen ME, Jaspers MW, van Strijp S, Fransen MP. Mobile Health for Smoking Cessation Among Disadvantaged Young Women During and After Pregnancy: User-Centered Design and Usability Study. JMIR formative research. 2021;5(8):e24112.
   Reason for exclusion: Ineligible participants.
5. De Wit-Zuurendonk LD, Oei S. Serious gaming in women's health care. BJOG. 2011;118(SUPPL. 3):17-21.
   Reason for exclusion: Ineligible participants.
6. Dininny JB. Food Rummy, the game of nutrition. MCN The American journal of maternal child nursing. 1977;2(2):90-1.
   Reason for exclusion: Ineligible intervention.
7. Ehlers DK, Huberty JL. Middle-aged women's preferred theory-based features in mobile physical activity applications. Journal of Physical Activity & Health. 2014;11(7):1379-85.
   Reason for exclusion: Ineligible intervention.
8. Font JM, Hedvall A, Svensson E, Acm. Towards Teaching Maternal Healthcare and Nutrition in Rural Ethiopia through a Serious Game. Extended Abstracts Publication of the Annual Sympsium on Computer-Human Interaction in Play (Chi Play'17 Extended Abstracts). 2017. p. 187-93.
   Reason for exclusion: Ineligible source.

9. Forsyth MC, Fulton DL, Lane DS, Burg MA, Krishna M. Changes in knowledge, attitudes and behavior of women participating in a community outreach education program on breast cancer screening. Patient Educ Couns. 1992;19(3):241-50.
Reason for exclusion: Ineligible intervention.
10. Gerling K, Hicks K, Buttrick L, Williams B, Hall J, Tang K, et al. Potential and Limitations of Playful Technology to Support Infant Feeding. Proceedings of the 2018 Annual Symposium on Computer-Human Interaction in Play Companion Extended Abstracts (Chi Play 2018). 2018. p. 431-7.
Reason for exclusion: Ineligible source.
11. Giunti G, Giunta DH, Guisado-Fernandez E, Bender JL, Fernandez-Luque L. A biopsy of Breast Cancer mobile applications: state of the practice review. Int J Med Inform. 2018;110:1-9.
Reason for exclusion: Ineligible intervention.
12. Gotlieb A, Louarn M, Nygard M, Ruiz-Lopez T, Sen S, Gori R, et al. Constraint-Based Verification of a Mobile App Game Designed for Nudging People to Attend Cancer Screening. Thirty-First AAAI Conference on Artificial Intelligence. 2017. p. 4678-85.
Reason for exclusion: Ineligible source.
13. Grassley JS, Connor KC, Bond L. Game-based online antenatal breastfeeding education: A pilot. Applied nursing research : ANR. 2017;33:93-5.
Reason for exclusion: Ineligible participants.
14. Hao HT, Dai XY. Research on Effect Evaluation of Health Game Participated by Occupational Women. Proceedings of Second International Conference on Sports Science and Sports Engineering. 2009. p. 267-71.
Reason for exclusion: Ineligible source.
15. Loihala M, Mustamu AC, Hasim NH. Effectiveness of Interactive Health Games on Knowledge, Attitude, Compliance with Immunization Officers and Family Planning. Pakistan Journal of Medical & Health Sceiences. 2020;14(2):1451-3.
Reason for exclusion: Ineligible participants.
16. Mueller S, Soriano D, Boscor A, Saville N, Arjyal A, Baral S, et al. MANTRA: development and localization of a mobile educational health game targeting low literacy players in low and middle income countries. BMC Public Health. 2020;20(1):1171.
Reason for exclusion: Ineligible participants.

17. Mueller S, Soriano D, Boscor A, Saville NM, Arjyal A, Baral S, et al. MANTRA: Improving Knowledge of Maternal Health, Neonatal Health, and Geohazards in Women in Rural Nepal Using a Mobile Serious Game. Frontiers in Public Health. 2020;8.
Reason for exclusion: Ineligible participants.
18. Musgrave LM, Homer CS, Kizirian NV, Gordon A. Identifying high quality mobile applications for pregnant women to improve outcomes for mothers and babies. J Paediatr Child Health. 2018;54(Supplement 1):37.
Reason for exclusion: Ineligible intervention.
19. Rosas LG, Trujillo C, Camacho J, Madrigal D, Bradman A, Eskenazi B. Acceptability of health information technology aimed at environmental health education in a prenatal clinic. Patient Educ Couns. 2014;97(2):244-7.
Reason for exclusion: Ineligible participants.
20. Rothwell E, Johnson E, Wong B, Rose NC, Latendresse G, Altizer R, et al. The Use of a Game-Based Decision Aid to Educate Pregnant Women about Prenatal Screening: A Randomized Controlled Study. Am J Perinatol. 2019;36(3):322-8.
Reason for exclusion: Ineligible participants.
21. Robertson MC, Baranowski T, Thompson D, Basen-Engquist KM, Swartz MC, Lyons EJ. Using the Behaviour Change Wheel Program Planning Model to Design Games for Health: Development Study. JMIR SERIOUS GAMES. 2021;9(4).
Reason for exclusion: Ineligible intervention.
22. Roubidoux MA. Breast cancer detective: a computer game to teach breast cancer screening to Native American patients. Journal of cancer education: the official journal of the American Association for Cancer Education. 2005;20(1 Suppl):87-91.
Reason for exclusion: Ineligible participants.
23. Sah YJ, Ratan R, Tsai H-YS, Peng W, Sarinopoulos I. Are you what your avatar eats? Health-behavior effects of avatar-manifested self-concept. Media Psychol. 2017;20(4):632-57.
Reason for exclusion: Ineligible participants.
24. Telfort J, Trivedi MS, Yi HS, Colbeth H, Vanegas A, Vargas J, et al. Implementing decision support for breast cancer chemoprevention in primary care. J Clin Oncol. 2017;35(15 Supplement 1).
Reason for exclusion: Ineligible source.
25. Thomas TH, Bender C, Rosenzweig M, et al. Testing the effects of the Strong Together self-advocacy serious game among women with advanced cancer: Protocol for the STRONG randomized clinical trial. *Contemporary clinical trials* 2023; 124. DOI: 10.1016/j.cct.2022.107003.

Reason for exclusion: Ineligible source.
