# Supplementary material for: Virtual serious games for women’s health education: A scoping review
Source: PLoS One. 2025 Jun 3;20(6):e0325327. doi: 10.1371/journal.pone.0325327 (PMC12133008; doi:10.1371/journal.pone.0325327)
Supplement: S3 File — (PDF) [file pone.0325327.s003.pdf]

S3 File. Data extraction tool

Author(s) (Year of Publication) Country of Origin	Research Aims/ Purpose	Methodology/ Study Design	Participants (Age and Sample Size)	Methods of Data Collection	Research Outcomes Assessed/ Measured	Target Area of Women's Health	Theoretical Framework	Engagement of Women From Target Group	Collaboration and Partnerships in VSG Development	Gaming Modality	Frequency and Length of Gameplay	Timing of Knowledge Post-Test After VSG
